# Illustrated keys to Scoliidae (Insecta, Hymenoptera, Scolioidea) from China

**DOI:** 10.3897/zookeys.1025.61385

**Published:** 2021-03-22

**Authors:** Zhen Liu, Cornelis Van Achterberg, Jun-Hua He, Xue-Xin Chen, Hua-Yan Chen

**Affiliations:** 1 College of Life and Environmental Sciences, Hunan University of Arts and Science, Changde 415000, China Hunan University of Arts and Science Changde China; 2 State Key Lab of Rice Biology, Ministry of Agriculture Key Lab of Molecular Biology of Crop Pathogens and Insects, Institute of Insect Sciences, Zhejiang University, Hangzhou 310058, China Zhejiang University Hangzhou China; 3 State Key Laboratory of Biocontrol, School of Life Sciences / School of Ecology, Sun Yat-sen University, Guangzhou 510275, China Sun Yat-sen University Guangzhou China

**Keywords:** China, illustrated keys, Scoliidae

## Abstract

The Scoliidae occur predominantly in tropical and subtropical regions and are ectoparasitoids of Scarabaeoidea larvae (especially of Melolonthinae) which are immobilised, parasitised by the female wasp in their terrestrial larval gallery and buried deeper in a special cell by the female wasp. Herein, we provided, for the first time, illustrated keys to 11 genera and 52 species of Scoliidae from China, based on specimens in the Naturalis Biodiversity Center (RMNH, Leiden) and additional specimens from the Chinese Academy of Insect Science (Beijing), Zhejiang University (ZJUH, Hangzhou) and Sun Yat-sen University (SYSU, Guangzhou) and it is a first attempt to make keys available for all the Scoliidae species in China.

## Introduction

Scoliid wasps (Hymenoptera, Scoliidae) form a medium-sized family with approx. 560 species worldwide and occur predominantly in tropical and subtropical regions (Osten 2005). Scoliidae parasitise exclusively second and third instar beetle larvae of Scarabaeoidea (Illingworth 1919, 1921). There are few studies on this group from China, though several species are common in south and central China. Fabricius (1781–1804) described 41 species of Scoliidae, including six from China. In the 18^th^ century, more than 20 species have been reported from China by several authors (Lepeletier 1845; Eversmann 1846, 1849; Smith 1855; de Saussure 1858; de Saussure et al. 1864; Morawitz 1889; Bingham 1896, 1897). In the 19^th^ century, Betrem (1928) published a monograph on the Indo-Australian Scoliidae, including 72 species and subspecies, of which 20 were reported from China. Later, Betrem (1941) published a systematic study on the Chinese Scoliidae including 2 genera, 61 species, subspecies and varieties from China, which is the first overview of the Chinese fauna, but partly confusing, because of naming too many (overlapping) subspecies and varieties. The systematics is in a state of confusion, which is well summarised by Elliott (2011). In China, 52 species are known (Liu et al., submitted), all belonging to the subfamily Scoliinae. For the present key, we examined Chinese specimens of four major collections: Naturalis Biodiversity Center (RMNH, Leiden, Netherlands), Chinese Academy of Insect science (IOZ, Beijing, China), Zhejiang University (ZJUH, Hangzhou, China) and Sun Yat-sen University (SYSU, Guangzhou, China). In this paper, we provide illustrated keys for all taxa of Scoliidae from China for the first time. This paper is part of an on-going project on the revision of the Chinese Scoliidae.

## Material and methods

This work is based on specimens in the collections of the Naturalis Biodiversity Center (**RMNH**), Chinese Academy of Insect Science (**IOZ**), Zhejiang University (**ZJUH**) and Sun Yat-sen University (**SYSU**).

Examination and measurements were made using a stereomicroscope (Zeiss Stereo Discovery V8). Detailed images of specimens were taken and processed using a digital camera Zeiss Stereo Discovery V12 and with the software Axiovision SE64 Rel.49.1. Habitus images were taken with a Nikon D600 camera coupled with a Nikon 60 mm Macro lens and processed with the software Combine ZM. All images were further processed using Adobe Photoshop CS5. Morphological terms for body structures and measurements followed Betrem et al. (1972). Wing venation nomenclature followed a modified version of the Comstock-Needham (van Achterberg 1979) and Betrem et al. (1972) system. Abbreviations used are as follows: POL = postocellar line, OOL = ocular-ocellar line, OD = ocellar diameter; T1–T6 = 1^st^ to 6^th^ tergite of metasoma; S1–S5 = 1^st^ to 5^th^ sternite of metasoma.

The genitalia were removed, cleaned and pinned under the specimen in a genitalia tube as described by Prosvirov and Savitsky (2011).

## Key to genera of the Scoliidae from China

**Table:** 

1	Second recurrent vein (2m-cu) of fore wing present **and** connected to second submarginal cell, resulting in two discal cells (a), **if** rarely absent (aa), **then** length of body 5–15 mm; mesopleural crest low, often carina-like, directed approximately to posterior corner of pronotum and with a small horizontal area at upper corner (b); T2 and T3 often with subapical row of setae bearing punctures (c); volsella of males divided into two parts (d); basal elevation of S3 (“gradulus”) or of T3 usually present (e); Campsomerini	**2**
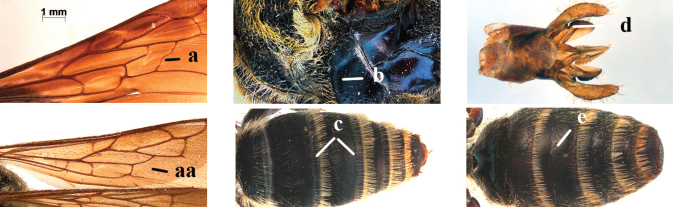		
–	Second recurrent vein (2m-cu) of fore wing absent, resulting in one discal cell (aa) and length of body more than15 mm **or** vein 2m-cu short and connected to first recurrent vein (1m-cu), thus resulting in a shorter second discal cell (aaa); mesopleural crest high, directed to the base of fore wing and with a large horizontal area (bb); T2 and T3 without subapical row of setae bearing punctures (cc); volsella of males not divided (dd); basal elevation of S3 or of T3 usually absent (ee); Scoliini	**12**
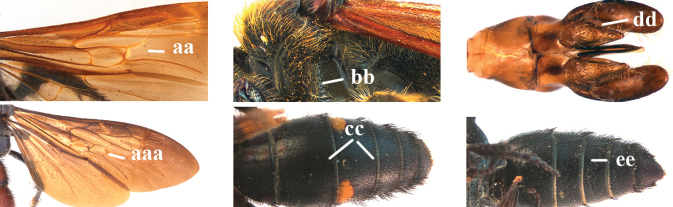		
2	Fore wing with 3 submarginal cells (a); T1–T4 often without subposterior transverse setose bands (b); hypostomal carina of male bisected, with submandibular triangle (c); ventral mandibular furrow of female exposed in frontal view (d); [“Trielidini/Colpinae auct.”; in China *C. tartara* (de Saussure, 1880)]	***Colpa* Dufour**
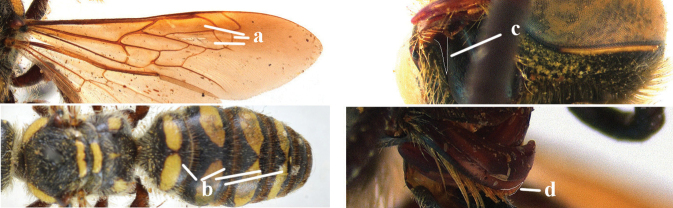		
–	Fore wing with 2 submarginal cells (aa); T1–T4 often with subposterior transverse setose bands (bb); hypostomal carina of male simple, without submandibular triangle (cc); ventral mandibular furrow of female concealed in frontal view (dd)	**3**
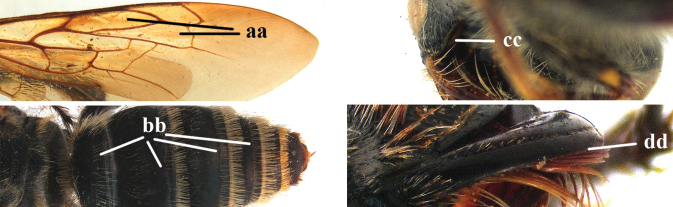		
3	Females (antenna with 12 segments)	**4**
–	Males (antenna with 13 segments)	**8**
4	Length of body 9–15 mm; base of hind wing without any carina below and upper plate of metapleuron smooth dorsally (a); metasomal tergites entirely black (b); metasomal setae white, except on apical tergite (c); wings subhyaline to pale brown (d)	***Micromeriella* Betrem**
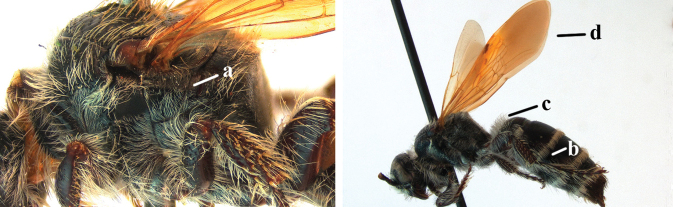		
–	Length of body 15–35 mm; base of hind wing with a carina below (aa) **or** upper plate of metapleuron more or less punctate dorsally, transition between its vertical and dorsal areas straight, with or without a carina below base of hind wing (aaa); metasomal tergites entirely black or with yellow or red pattern (bb); metasomal setae often differing in colour from the tegument (cc); wings subhyaline to dark brown (dd)	**5**
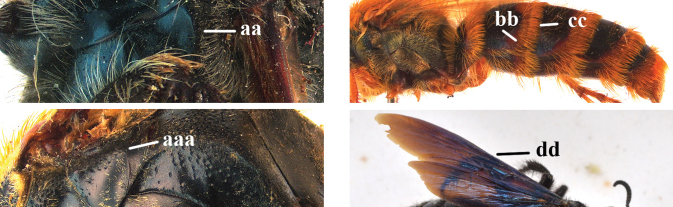		
5	Upper plate of metapleuron smooth, sometimes with fine, sparse punctures dorsally, transition between its vertical and dorsal areas straight and with an entirely or partially distinct carina below base of hind wing (a)	**6**
–	Upper plate of metapleuron punctate dorsally, transition between its vertical and dorsal areas usually straight anteriorly and gradually sloping posteriorly and without a distinct carina below base of hind wing (aa)	**7**
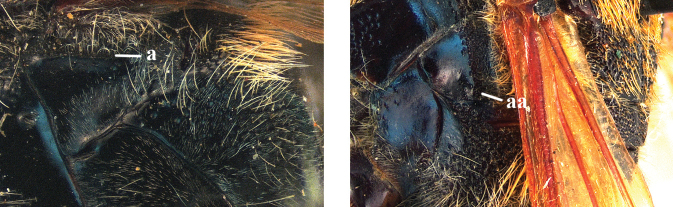		
6	Lateral carina of propodeum at most extending up to level of spiracle (a); dorsal area of propodeum triangularly protruding medio-posteriorly and its posterior surface impunctate to remotely punctate (b); vertex behind posterior ocelli impunctate (c); body black, 20–30 mm long (d); wings entirely fuscous to yellowish subhyaline (e)	***Campsomeriella* Betrem**
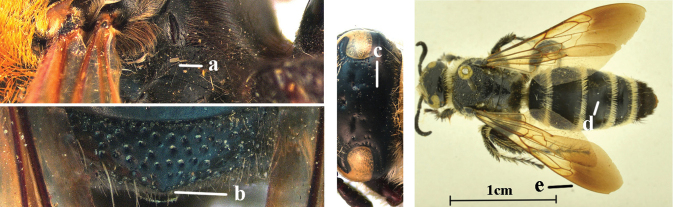		
–	Lateral carina of propodeum extended beyond level of spiracle (aa); dorsal area of propodeum truncated medio-posteriorly and its posterior surface densely punctate, at least dorsally (bb); vertex behind posterior ocelli with sparse to dense punctation (cc); metasoma partly yellow or red, body length more than 30 mm (dd); wings dark brown (ee)	***Sericocampsomeris* Betrem**
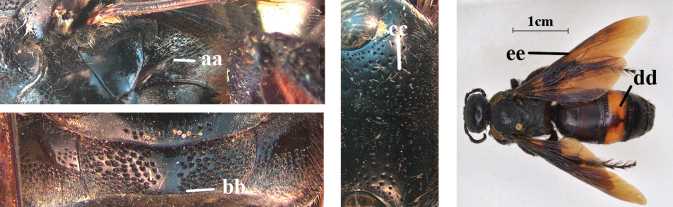		
7	Frons with cluster of deep punctures in front of anterior ocellus (a); first submarginal cell of fore wing almost entirely setose (b); fore wing dark brown subapically (c)	***Phalerimeris* Betrem**
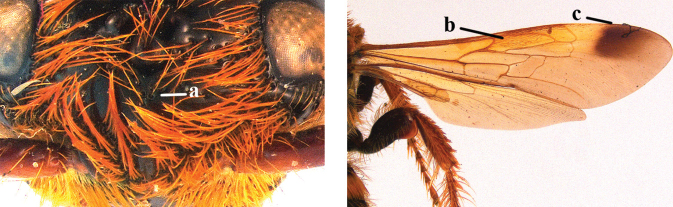		
–	Frons usually without deep punctures in front of anterior ocellus (aa); first submarginal cell fore wing glabrous, except for some setae anteriorly (bb); fore wing at most indistinctly smoky subapically (cc)	***Megacampsomeris* Betrem**
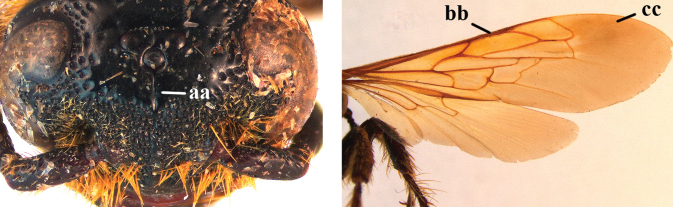		
8	Length of body 5–15 mm; frontal spatium sparsely punctate, intervals between punctures larger than puncture diameters, especially medially (a); scutellum and metanotum usually marked with yellow spots; T1 always with yellow apical band which usually narrowed medially (b); parameres more slender, 4.6× longer than wide (c). [Note: As far as is known, vein 2m-cu of fore wing is always developed in Chinese species, but this vein is occasionally reduced or absent in some species]	***Micromeriella* Betrem**
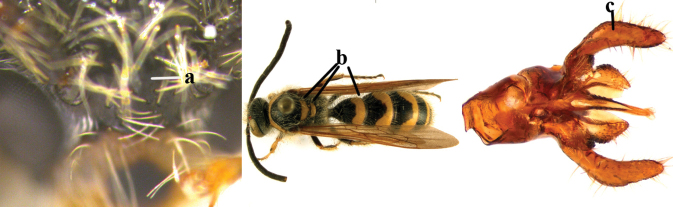		
–	Length of body 20–30 mm; frontal spatium with dense punctation, intervals mostly smaller than puncture diameter (aa); yellow spots absent or present on scutellum and metanotum and shape of yellow apical band of first tergite variable (bb); parameres robust, 3.2× longer than wide (cc)	**9**
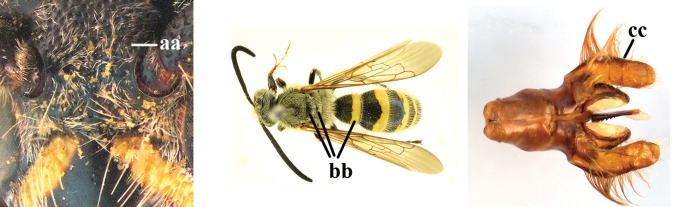		
9	Head distinctly broader than high in frontal view (width approx. 1.3× height) (a); frons largely impunctate and with a large, flat, triangular area in front of anterior ocellus (b); apical metasomal sternites usually with dense erect setae (copulatory brushes) (c); parameres distinctly angled in middle, not narrowed apicad, basal part of volsellae lacking dense setae (d)	***Campsomeriella* Beterem**
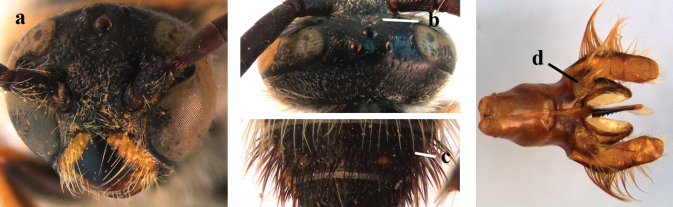		
–	Head almost as broad as high in frontal view (width at most 1.1× height) (aa); frons without a large impunctate area or triangular area in front of anterior ocellus (bb); apical metasomal sternites without dense erect setae (cc); parameres usually rounded in middle, narrower apicad, basal part of volsellae with sparse to dense setae (dd)	**10**
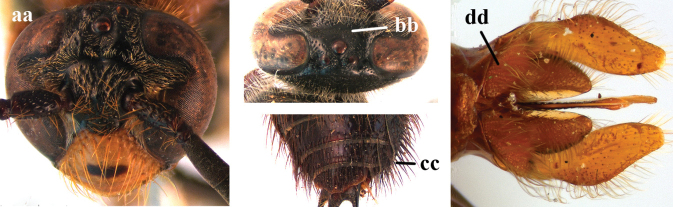		
10	Clypeus, mandible, scapula, scutellum and metanotum entirely black, metasoma usually with wide reddish or orange bands on T1–3, often also on apical tergites (a); hind tibial spurs black (b); parameres less slightly converging apically (c)	***Sericocampsomeris* Betrem**
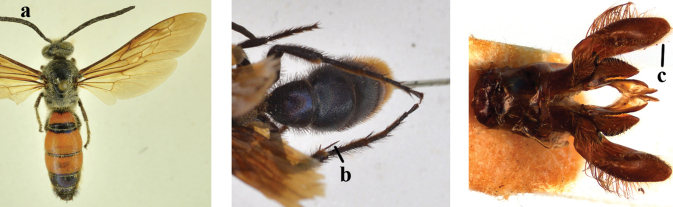		
–	Clypeus, mandible, scapula, scutellum or metanotum often partly yellow, metasoma usually with narrow yellowish to reddish bands at least on basal four tergites (aa); hind tibial spurs white to pale yellowish (bb); parameres strongly converging apically (cc)	**11**
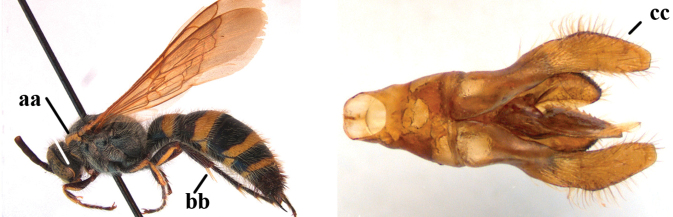		
11	Basal part of volsellae with short sparse setae, distance between bases of these setae more than their own diameter (a); length of body less than 14 mm	***Phalerimeris* Betrem**
–	Basal part of volsellae with long dense setae, distance between the bases of these setae less than their own diameter (aa); length of body more than 15 mm, often 20–30 mm	***Megacampsomeris* Betrem**
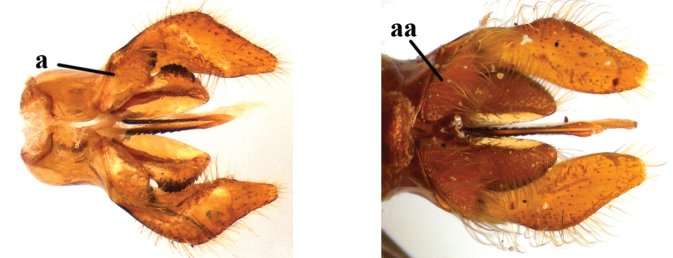		
12	Fore wing with two discal cells (a)	***Liacos* Guérin-Méneville**
–	Fore wing with one discal cell (aa)	**13**
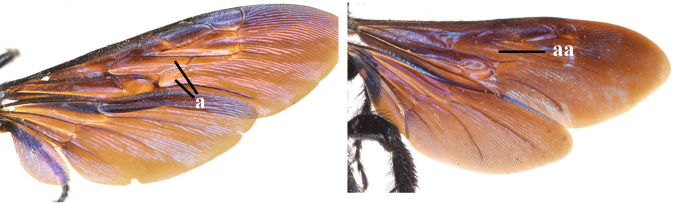		
13	Fore wing with three submarginal cells (a)	**14**
–	Fore wing with two submarginal cells (aa)	**15**
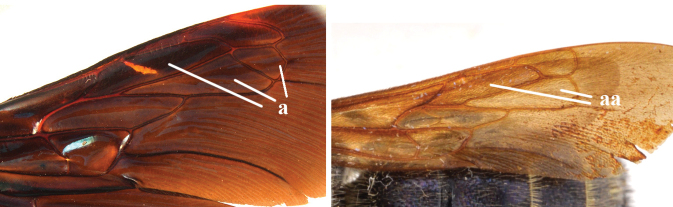		
14	Length of body 15–20 mm; frons with an arched ridge, running from one ocular sinus to another and passing behind anterior ocellus, ridge prominent in male (a), but rather weak in female; ocelli on high triangle, posterior tangent to anterior ocellus far away from posterior ocelli in both sexes (b); metasomal setae black (c); T1 without tubercle (d); [only *Austroscolia ruficeps* (Smith, 1855) occurs in China]	***Austroscolia* Betrem**
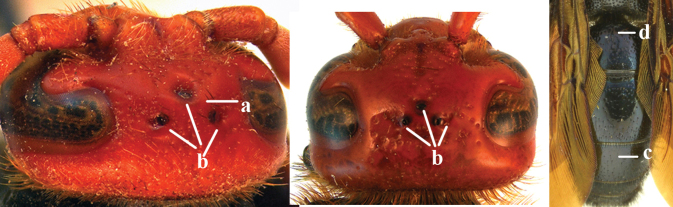		
–	Length of body 23–42 mm; frons without an arched ridge (aa); ocelli on low triangle, posterior tangent to anterior ocellus nearly touching posterior ocelli in both sexes (bb); setae of T3–6 or T4–6 reddish (cc); T1 with a strong tubercle antero-medially (dd); [only Megascolia (Regiscolia) azurea (Christ, 1791) occurs in China]	***Megascolia* Betrem**
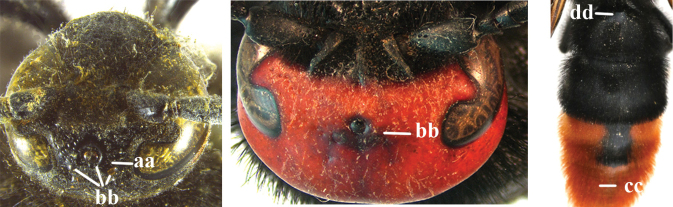		
15	Frons often with a transverse ridge (or carina) on the males (a); posteriorly not strongly sloping vertex and temples less receding dorsally (b); metasoma black (c), at most T3 with yellow spots (cc), often with purple and blue reflections	***Carinoscolia* Betrem**
–	Frons without transverse carina on the males (aa); vertex strongly sloping posteriorly and temples more receding dorsally (bb); metasoma often with extensive yellow pattern (ccc) and with variable reflections	***Scolia* Fabricius**
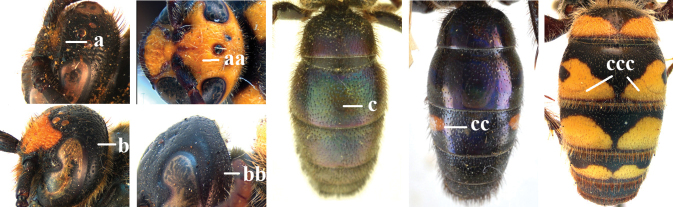		

### Key to species of genus *Campsomeriella* from China

**Table:** 

1	Females, antenna with 12 segments (female of *C. ilanensis* (Tsuneki, 1972) unknown)	**2**
–	Males, antenna with 13 segments	**5**
2	Lateral carina of propodeum short, not reaching spiracles (a); spurs of hind tibia black or testaceous (b); setae yellow to reddish on head and mesosoma (c); subgenus Campsomeriella Betrem	**3**
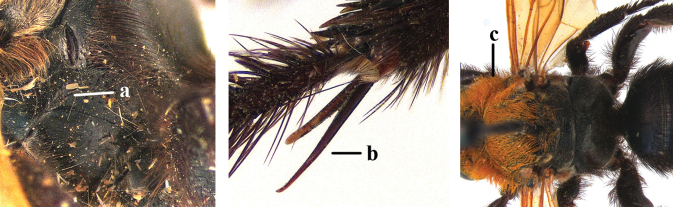		
–	Lateral carina of propodeum long, extending somewhat beyond the spiracles (aa); spurs of hind tibia white (bb); setae white on head and thorax (cc); subgenus Annulimeris Betrem	**C. (A.) annulata (Fabricius 1793)**
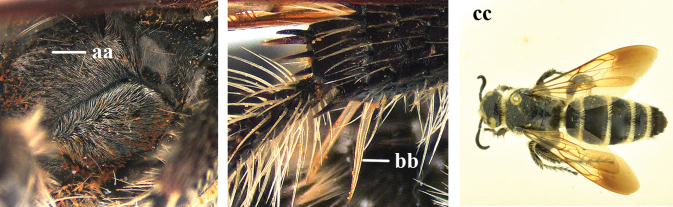		
3	Setae of metasoma entirely black (a); setae on vertex and pronotum red (b); wings distinctly darkened apically (c), sometimes also basally and medially (cc)	**4**
–	Fringes of T1–T5 white (aa); setae on vertex and pronotum yellow (bb); wings subhyaline, at most, faintly darkened anteriorly (ccc)	**C. (C.) sauteri (Betrem, 1928)**
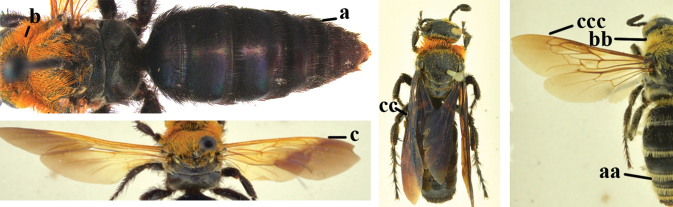		
4	Wings with distinctly darkened apical third and remainder subhyaline (a); vertex sparsely and distinctly punctate, densely pubescent (b); disc of clypeus with longitudinal ridge medially (c)	**C. (C.) thoracica (Fabricius, 1787)**
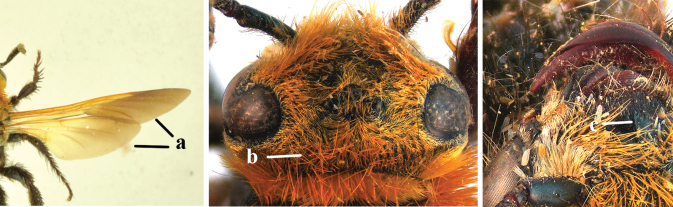		
–	Wings largely dark brown (aa); vertex smooth, except near occipital carina; disc of clypeus without ridge (cc); [syn. with *C.* (?) *ilanensis* (Tsuneki, 1972)]	**C. (C.) collaris (Fabricius, 1798)**
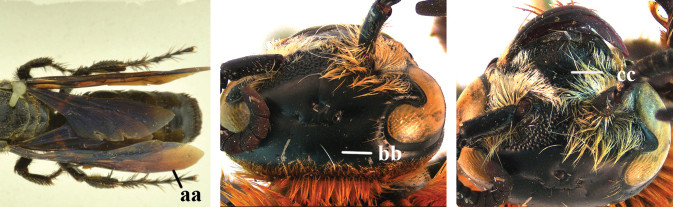		
5	Base of volsella covered with sparse and short setae (a); metanotum black (b) and scutellum sometimes with two yellow spots; subgenus Campsomeriella Betrem	**6**
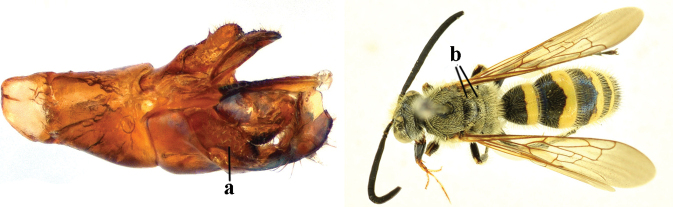		
–	Base of volsella covered with long and dense setae (aa); metanotum and scutellum always with yellow spots (bb); subgenus Annulimeris Betrem	**C. (A.) annulata (Fabricius 1793)**
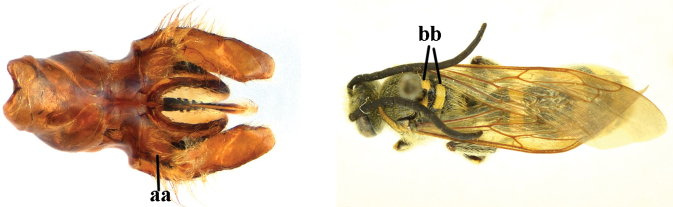		
6	Bands of apical metasoma tergites red or orange, wide extending over half of tergites surface; coloured bands often absent on T1 (a); reddish setae covering most of metasoma coloured bands (b)	**C. (C.) thoracica (Fabricius, 1787)**
–	Bands of apical metasoma segments yellow and often narrow, at most covering half of the tergites surface; T1 always with yellow apical band (aa); white setae covering yellow bands; brown to blackish-brown setae on T7 or T5–7 and T1–T6 or T1–T4 with white setae (bb)	**7**
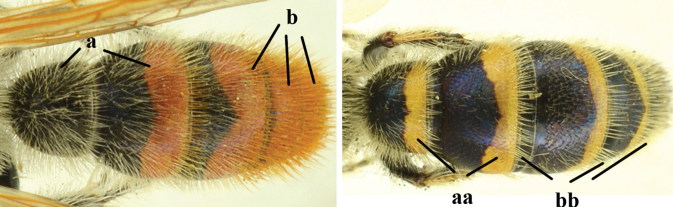		
7	Pronotum yellow posterodorsally (a); middle femur marked with yellow apically; hind tibia extensively yellow (b); yellow band on T3 covering nearly half of its mid-length (c); [syn. with *C.* (?) *ilanensis* (Tsuneki, 1972)]	**C. (C.) collaris (Fabricius, 1798**
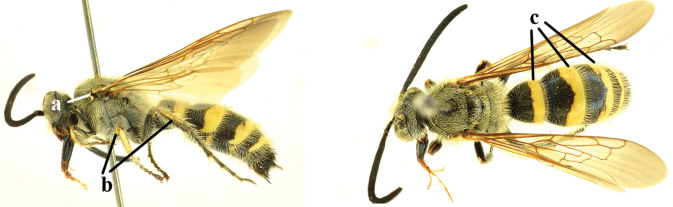		
–	Pronotum black posterodorsally (aa); middle femur nearly entirely black; hind tibia yellow basally (bb); yellow band on T3 occupying 1/4 of its mid-length (cc)	**C. (C.) sauteri (Betrem, 1928)**
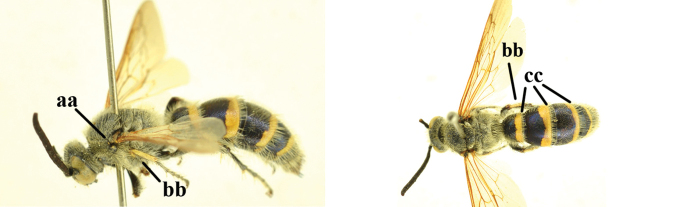		

**Notes.** Only the male holotype of *Campsomeriella ilanensis* from Taiwan is known. According to the description and drawings by Tsuneki (1972a), *C. ilanensis* is very similar to C. (C.) collaris quadrifasciata (Fabricius, 1798) and it could be a synonym of the latter.

### Key to species of *Megacampsomeris* from China

**Table:** 

1	Female, antenna with 12 segments (female of *M. szetschwanensis* (Betrem, 1928) unknown)	**2**
–	Male, antenna with 13 segments (males of *M. bella* (Bingham, 1897), *M. grossa* (Fabricius, 1804) and *M. stoetzneri* (Betrem, 1928) unknown)	**14**
2	Fringes of T1–T3 white or whitish (a)	**3**
–	Fringes of T1–T3 yellow, reddish-yellow or black (aa)	**8**
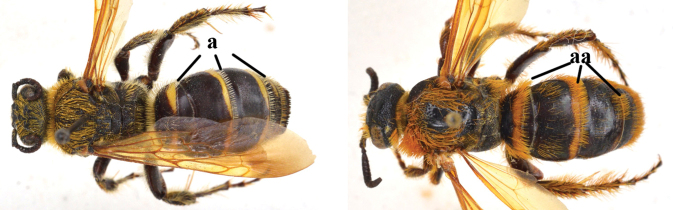		
3	At least T1 more or less yellow or pale yellow posteriorly (a)	**4**
–	Metasomal tergites completely black (aa)	**6**
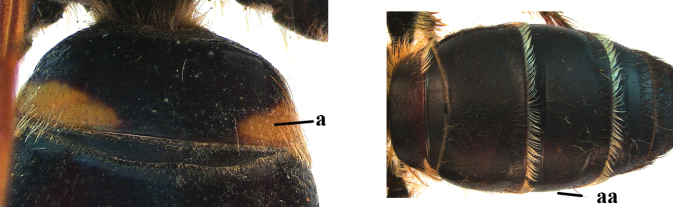		
4	Vertex, behind ocelli, largely smooth (a); frons, in front of ocelli, narrowly smooth (b); mesoscutum smooth medially (c)	***M. schulthessi* (Betrem, 1928)**
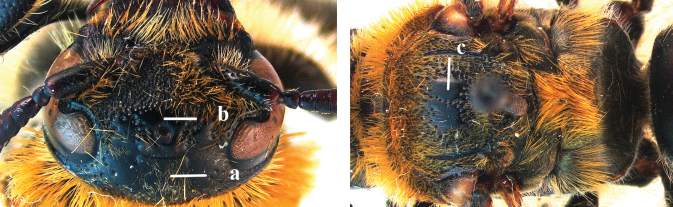		
–	Vertex (aa) and frons (bb) largely, densely and deeply punctate; mesoscutum entirely densely punctate (cc)	**5**
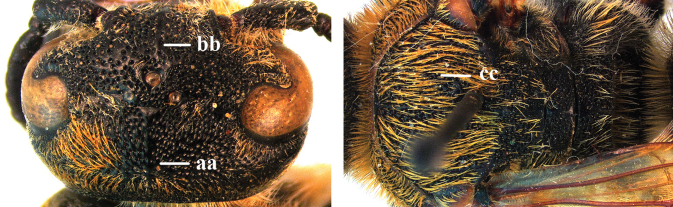		
5	Spiracular corner (a) and vertical portion of upper plate of metapleuron (b) impunctate; upper plate of metapleuron divided by a well-defined ridge into a punctate dorsal portion and an impunctate vertical portion (c)	***M. bella* (Bingham, 1897)**
–	Spiracular corner punctate (aa); vertical portion of upper plate of metapleuron punctate just below transition from dorsal portion and with few punctures along anterior and posterior sutures (bb); transition from dorsal to vertical portion of upper plate of metapleuron gradual, not carina-like (cc)	***M. stoetzneri* (Betrem, 1928)**
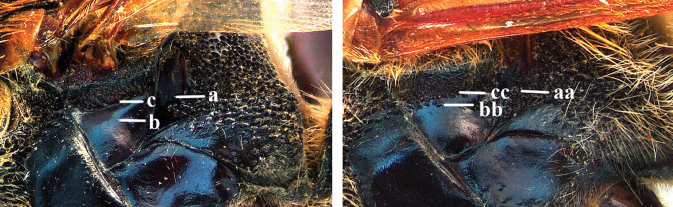		
6	Vertex entirely smooth behind ocelli (a); setae blackish above antennal insertion and along inner margin of eyes (b)	***M. grossiformis* Betrem, 1928**
–	Vertex densely punctate behind ocelli (aa); head setae yellow or yellowish-brown (bb)	**7**
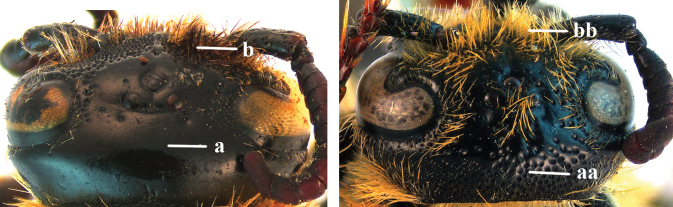		
7	Mesoscutum and scutellum smooth, except for few punctures laterally (a); metanotum broadly smooth medially (b)	***M. farrenwhitei* Betrem, 1928**
–	Mesoscutum and scutellum nearly entirely densely punctate, except for more or less smooth area posteromedially (aa); metanotum densely punctate (bb)	***M. grossa* (Fabricius, 1804)**
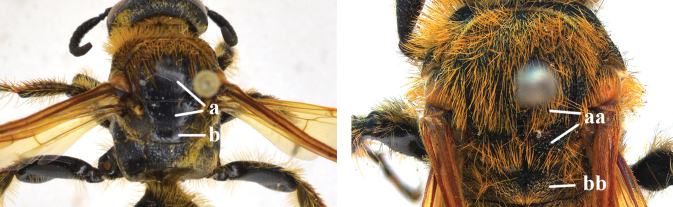		
8	Fringes of T1–T4 blackish-brown to black; wings distinctly darkened	***M. binghami* (Betrem, 1928)**
–	Fringes of T1–T4 brown or yellowish-brown (a); wings subhyaline and yellowish (b)	**9**
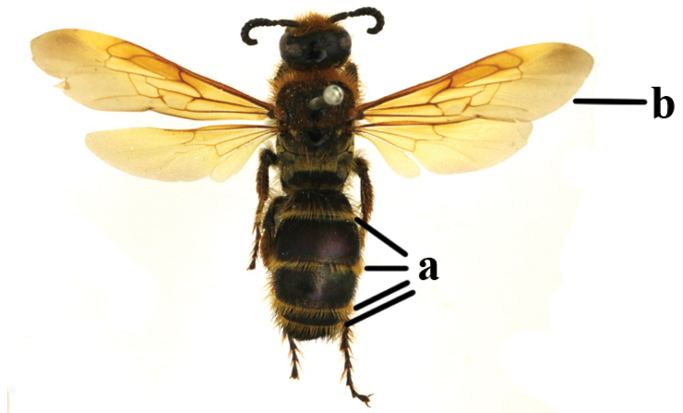		
9	T1 and T2, sometimes including T3, with a yellow or pale yellow band posteriorly (a)	**10**
–	Tergites entirely black (aa)	**11**
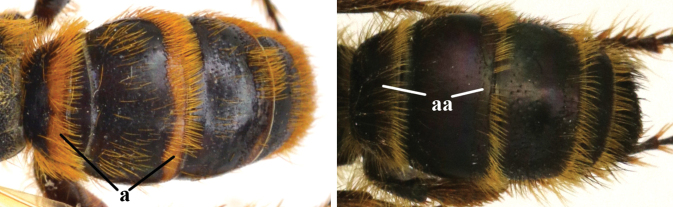		
10	Bands on tergites distinct, T3 usually with yellow area posteriorly (a); scutellum largely smooth medially (b); basal elevation of T2 (“gradulus”) distinctly developed (c)	***M. formosensis* Betrem, 1928**
–	Bands on tergites indistinct, T3 without yellow area (aa); scutellum evenly and densely punctate (bb); basal elevation of T2 absent (cc)	***M. limbata* (de Saussure & Sichel, 1864)**
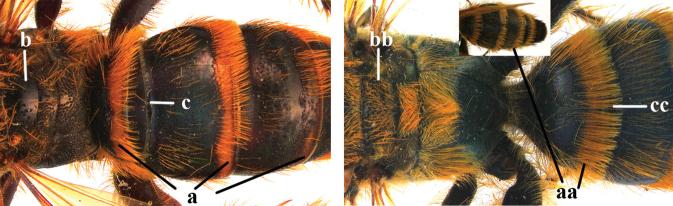		
11	Vertex smooth latero-posteriorly (a)	***M. lindenii* (Lepeletier, 1845)**
–	Vertex coarsely punctate latero-posteriorly (aa)	**12**
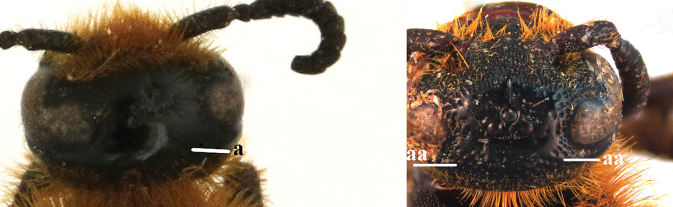		
12	Scutellum largely smooth (a); wings without infuscation subapically (b); median groove of frons distinct (c)	***M. farrenwhitei* Betrem, 1928**
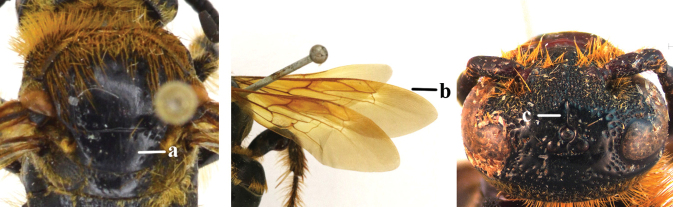		
–	Scutellum punctate anteriorly (aa), **if** largely smooth, then wings with subapical infuscation (bb); median groove of frons partly or almost completely missing (cc)	**13**
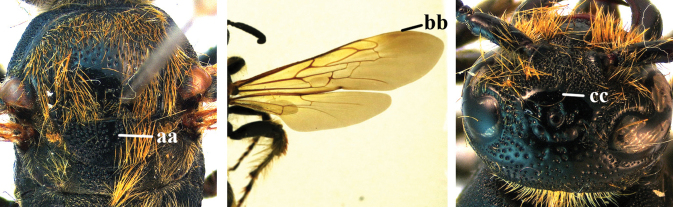		
13	Fringes of T1–T4 yellowish-brown and black on other tergites (a); antenna black (b); scutellum entirely or only anteriorly punctate (c)	***M. prismatica* (Smith, 1855)**
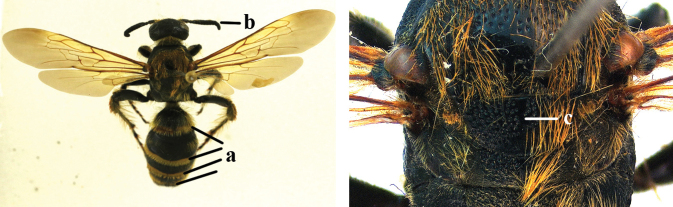		
–	Tergites setae yellowish-brown; antenna reddish-yellow; scutellum largely smooth	***M. ceylonica* (Kirby, 1889)**
14	Tergites setae red or reddish-brown (a); legs yellowish-brown (b)	***M. ceylonica* (Kirby, 1889)**
–	Setae pale yellowish (aa) or reddish-brown and, on T6–T7, black (aaa); legs mainly black (bb)	**15**
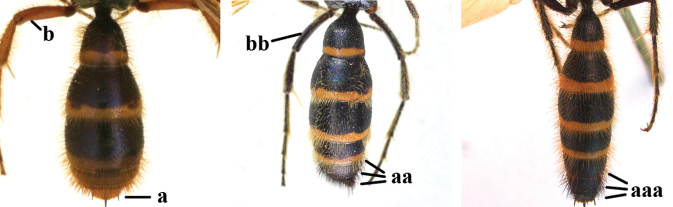		
15	Clypeus mostly black, at most with two small lateral yellowish areas above mandible base	**16**
–	Clypeus at least broadly yellow laterally (a)	**19**
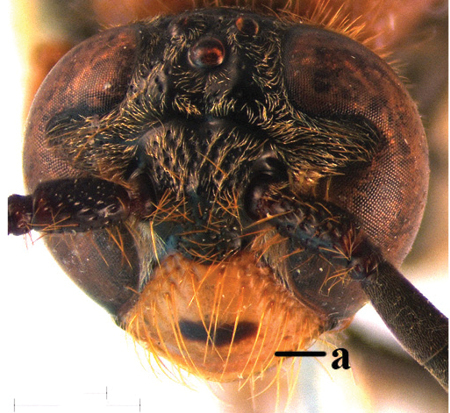		
16	Wings more or less darkened (a)	***M. binghami* (Betrem, 1928)**
–	Wings subhyaline (aa)	**17**
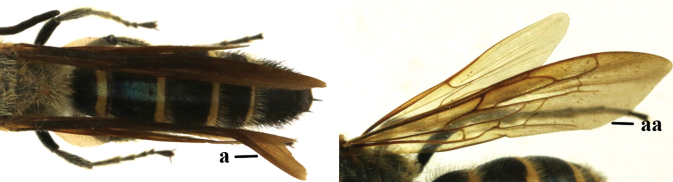		
17	Posterodorsal margin of pronotum pale yellow; scutellum without medio-longitudinal carina	***M. schulthessi* (Betrem, 1928)**
–	Posterodorsal margin of pronotum only indistinctly narrowly yellow posteriorly (a); scutellum and metanotum with a more or less distinct medio-longitudinal carina (b)	**18**
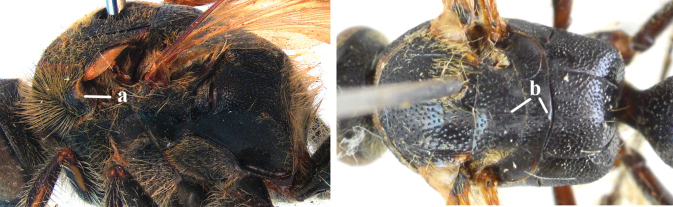		
18	Setae on mesosoma yellowish and sparse (a); punctures sparse, intervals 1–2× larger than puncture diameter (b)	***M. formosensis* (Betrem, 1928)**
–	Setae on mesosoma reddish and dense (aa); punctures dense, intervals often smaller than puncture diameter (bb)	***M. szetschwanensis* (Betrem, 1928)**
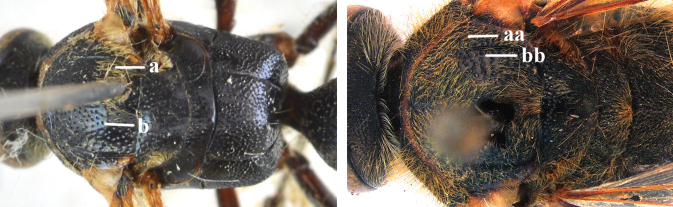		
19	Clypeus always black medio-ventrally and remainder more or less yellow (a)	**20**
–	Clypeus always yellow medially (aa)	**22**
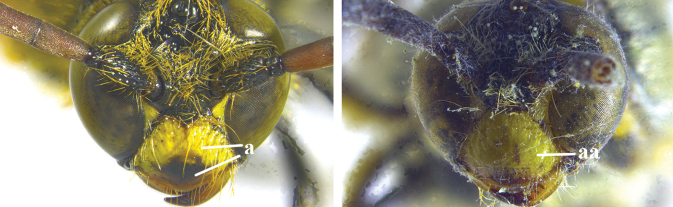		
20	Yellow bands on T1 wide, covering nearly 1/3 of T1; scutellum always with two lateral yellow spots	***M. limbata* (de Saussure & Sichel, 1864)**
–	Yellow bands on T1 narrow, at most covering 1/5 of T1; scutellum often entirely black, rarely with yellow spots	**21**
21	Hind femur yellow ventrally (a); S2–S4 often with yellow cuneate maculae lateroapically (b)	***M. farrenwhitei* Betrem, 1928**
–	Hind femur black ventrally (aa); S2–S4 rarely with yellow maculae (bb)	***M. prismatica* (Smith, 1855)**
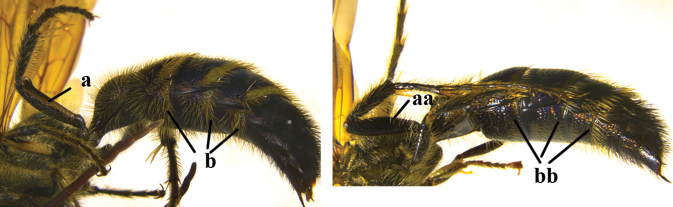		
22	T5 usually with yellow band posteriorly; setae of mesosoma dirty white; pronotum black	***M. grossiformis* Betrem, 1928**
–	T5 without a yellow band (a); setae of mesosoma reddish or yellowish-brown (b); pronotum entirely or mostly reddish-yellow (c)	***M. lindenii* (Lepeletier, 1845)**
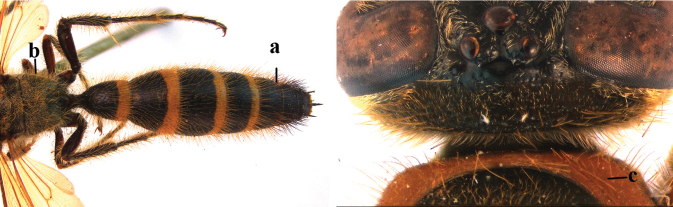		

**Notes.** Males of this genus are difficult to recognise, the key above is a practical key to the males present in the material we have studied and is just a first attempt. The male of *M. farrenwhitei* Betrem, 1928 is here recorded for the first time worldwide. However, males identified as *M. szetschwanensis* (Betrem, 1928) could be the male of *M. stoetzneri* Betrem or another species, since the female of *M. szetschwanensis* and the males of *M. bella* (Bingham, 1897), *M. grossa* (Fabricius, 1804) and *M. stoetzneri* (Betrem, 1928) are unknown and the forma A (in Betrem 1941, typical forma of *C. szetschwanensis*) specimen of *M. szetschwanensis* carries an identical collection label as the holotype female of *M. stoetzneri* (Schulten 2011).

### Key to species of *Sericocampsomeris*

**Table:** 

1	Female, antenna with 12 segments	**2**
–	Male, antenna with 13 segments	**3**
2	Pronotum posterodorsally and upper margin of clypeus with golden setae (a); metasoma setae dark brown, except indistinctly reddish-brown setae on epipygium (b); metanotum and propodeum densely punctate (c)	***S. degaullei* (Betrem, 1928)**
–	Pronotum posterodorsally and clypeus with black setae (aa); setae on T2 or T5–T7 reddish-golden (bb); metanotum and median part of propodeum largely smooth (cc)	***S. rubromaculata* (Smith, 1855)**
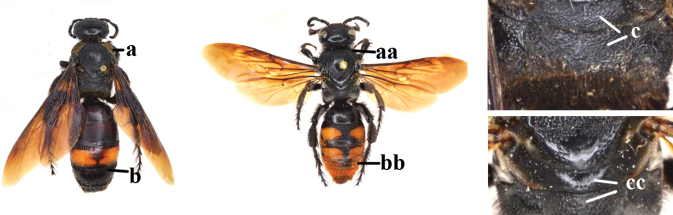		
3	Posterior part of T3 and T4–T7 bright reddish-yellow (a); wings infuscate (b); scutellum and metanotum with a continuous medio-longitudinal carina (c)	***S. degaullei* (Betrem, 1928)**
–	T1–T3 predominantly reddish-yellow with pale yellow setae, except for black setae on T5/T6–T7 (aa); wings hyaline (bb); scutellum and metanotum without medio-longitudinal carina (cc)	***S. rubromaculata* (Smith, 1855)**
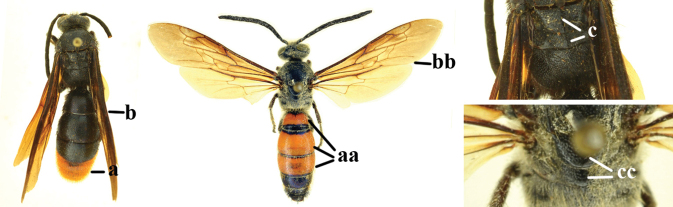		

### Key to species of *Carinoscolia* Betrem from China

**Table:** 

1	Frons with a distinct transverse carina present in front of anterior ocellus in both sexes (a); head dark in female (b)	***C. nipponensis* Uchida, 1933**
–	Frons without transverse carina, at most with distinct transverse ridge present in males (aa), but often with more or less denser punctate depression before a relatively higher area in both sexes (aaa); head with more or less yellow areas (bb)	**2**
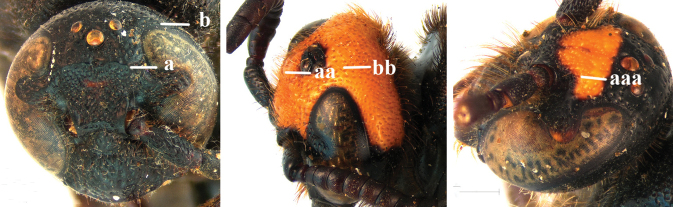		
2	Frons with a distinct transverse ridge in male (a); pronotum in both sexes yellow laterally (b); T3 and sometimes also T4 in female yellow laterally (c), male metasoma wholly black	***C. yunnanensis* (Betrem, 1941)**
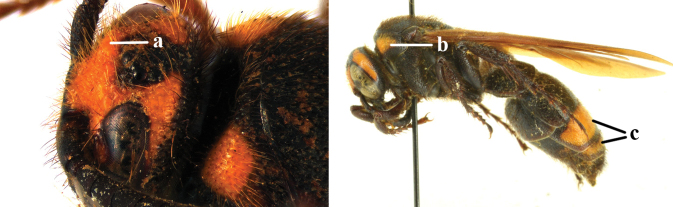		
–	Frons without distinct transverse ridge in male, often replaced by a more or less denser punctate depression (aa); pronotum in both sexes black laterally (rarely with small round yellow spot anteriorly in female); T3 of both sexes often with pale yellow lateral spot (cc)	***C. vittifrons* (Sichel, 1864)**
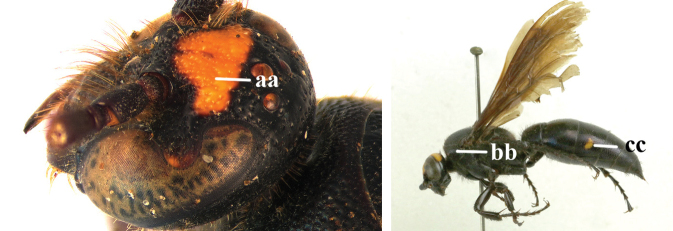		

### Key to species of the genus *Scolia* Fabricius from China

**Table:** 

1	Base of volsella covered with a dense brush of silky setae (a); Chinese female either with metasoma entirely black and wings evenly infuscate, lacking purple or blue reflections (b) **or** with metasoma predominantly yellow and wings bright yellow basally (bb); mainly distributed in Palaearctic Regions of China; subgenus Scolia s. str. Fabricius	**2**
–	Base of volsella without dense brush of silky setae (aa); females either with metasoma predominantly black and wings dark brown with distinct purple or blue reflections (bbbb) **or** with red or yellow pattern and wings never bright yellow basally (bbb); mainly distributed in Oriental Regions of China; subgenus Discolia Saussure	**4**
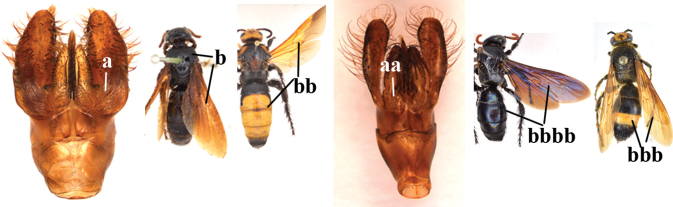		
2	Body black (a); wings not distinctly darkened apically and evenly infuscate (b); clypeus nearly smooth medially (c)	**S. (S.) concolor Eversmann, 1849**
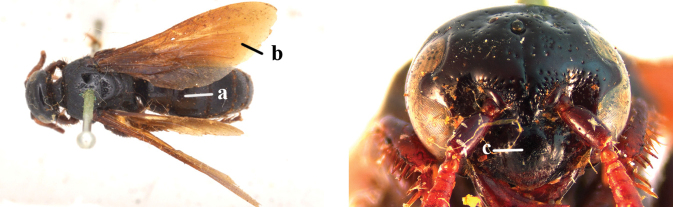		
–	Metasoma at least partly yellowish (aa); wings with distinct dark apical band and yellowish basally (bb); clypeus strongly rugose or densely punctate medially (cc)	**3**
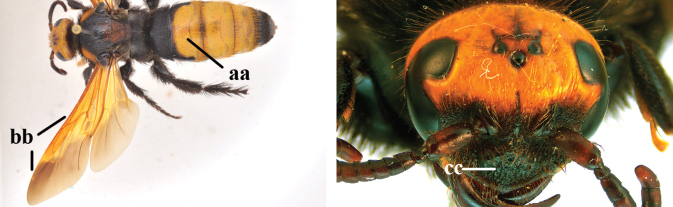		
3	Vertex smooth behind ocelli (a); anterior ocellus situated in a narrow and shallow depression (b); vertex and frons yellow (c)	**S. (S.) flaviceps Eversmann, 1846**
–	Vertex densely punctate (aa); anterior ocellus in a broad and deep depression (bb); vertex and frons black (cc)	**S. (S.) potanini Morawitz, 1889**
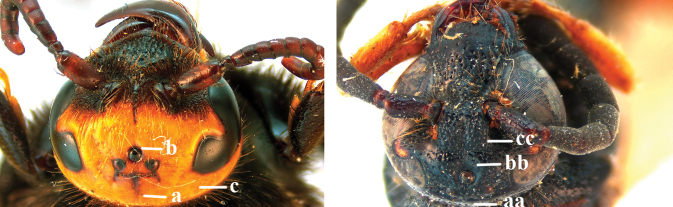		
4	Setae red or yellowish-brown on T2–T7 (sometimes black on epipygium) and black on mesosoma (a)	**5**
–	Setae on T4–T7 usually black (aa), **if** setae pale, **then** mesosoma setae also pale	**6**
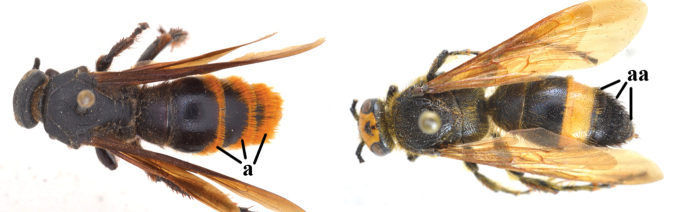		
5	Body entirely black, setae red on T2–T7 (a); fore wing without dark subapical spot (b); mesopleuron densely punctate, except partly anteriorly and posteriorly (c)	**S. (D.) sinensis de Saussure & Sichel, 1864**
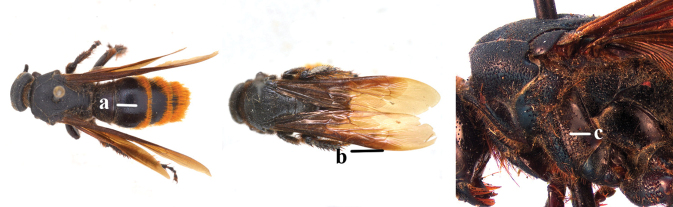		
–	T3–T6 largely reddish-yellow with red setae, fringes of T2 black; fore wing with a darker subapical spot; mesopleuron nearly smooth, with a few large punctures	**S. (D.) minowai Uchida, 1933**
6	Female, antenna with 12 segments (females of S. (D.) bnun Tsuneki, 1972 and S. (D.) wusheensis Tsuneki, 1972 unknown)	**7**
–	Male, antenna with 13 segments (males of S. (D.) inouyei Okamoto, 1924 and S. (D.) tigrimaculosa Yamane, 1995 unknown)	**23**
7	Dorso-median area of propodeum smooth to superficially and sparsely punctate (intervals at least as large as puncture diameter) (a)	**8**
–	Dorso-median area of propodeum more or less strongly and densely punctate (intervals smaller than puncture diameter) (aa)	**11**
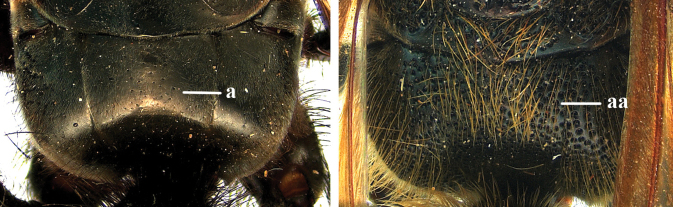		
8	Metasoma and mesosoma completely black (a)	**9**
–	Metasoma and/or mesosoma often with yellow pattern (aa)	**10**
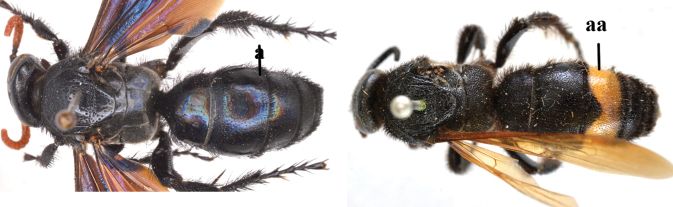		
9	Vertex partially to almost entirely yellowish-red (a); metanotum with few, irregular punctures (b); POL:OD:OOL=183:54:251 (c); wings without reflection (d)	**S. (D.) superciliaris de Saussure, 1864**
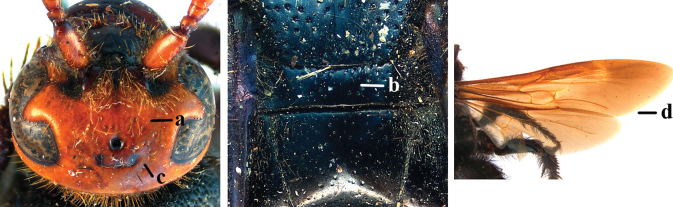		
–	Vertex entirely black (aa); metanotum and propodeal dorsally almost entirely smooth (bb); POL:OD:OOL=153:69:183 (cc); wings with distinct purple reflection (dd)	**S. (D.) affinis Guérin-Méneville, 1845**
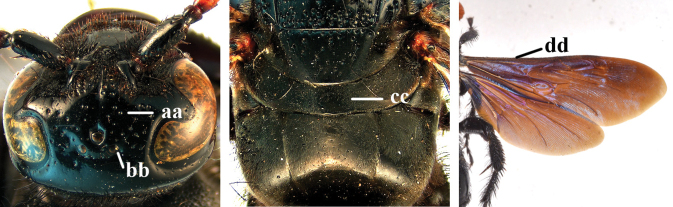		
10	T4 black (a); lateral carina just reaching level of propodeal spiracle (b); median groove of frons distinct (c)	**S. (D.) nobilis de Saussure, 1858**
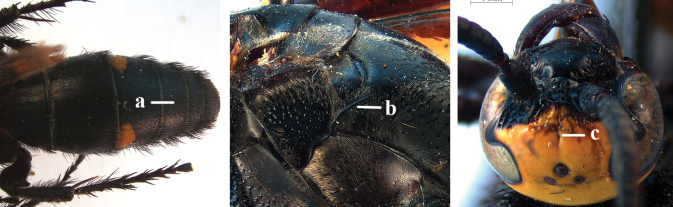		
–	T4 with a narrow apical yellow band or two lateral yellow spots (aa); lateral carina surpassing level of propodeal spiracle (bb); median groove of frons absent (cc)	**S. (D.) inouyei Okamoto, 1924**
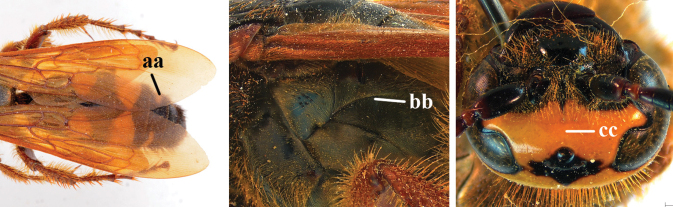		
11	Body black (a)	**S. (D.) laeviceps Smith, 1855**
–	Metasoma often with red or yellow pattern (aa)	**12**
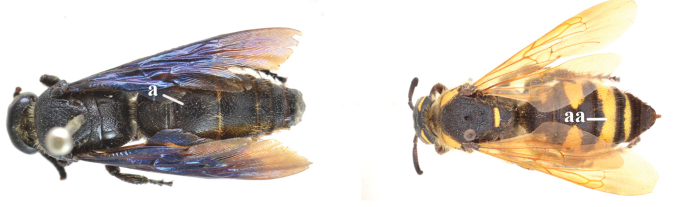		
12	Pronotum posterodorsally yellow or red (a)	**13**
–	Pronotum posterodorsally black (aa)	**17**
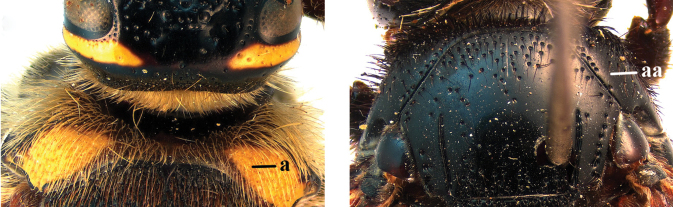		
13	Middle of clypeus and anterior part of head above antenna sockets red; pronotum marked with red	**S. (D.) clypeata Sickman, 1894**
–	Head black or only with yellow spots; pronotum marked with yellow	**14**
14	Mesonotum often evenly densely punctate (a); spiracular corner often smooth (b); pronotum posterodorsally widely smooth (c); scutellum and/or metanotum always with yellow bands (d)	**S. (D.) picteti de Saussure, 1855)**
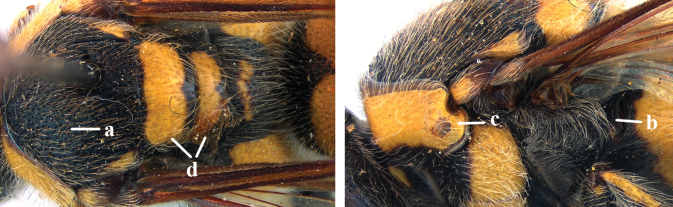		
–	Mesonotum narrowly to largely impunctate medially; spiracular corner punctate, **if** smooth, **then** pronotum posterodorsally largely sculptured; pale pattern on scutellum and metanotum less developed	**15**
15	Area behind hind ocellus almost impunctate; gena with a yellow line behind eye	**16**
–	Area behind hind ocellus with well-defined punctures; gena without yellow line	**S. (D.) desidiosa Bingham, 1896**
16	Scutellum black; anterior half of mesopleuron almost impunctate; apical fringe of T2–4 and S5 dark brown	**S. (D.) taiwana Tsuneki, 1972**
–	Scutellum largely yellow; anterior half of mesopleuron extensively closely punctate; apical fringe of T2–4 and S5 pale golden	**S. (D.) tigrimaculosa Yamane, 1995**
17	Legs reddish (a); wings yellow hyaline, with dark tip (b); metasoma with reddish-brown setae throughout (c); T2–5 with yellow apical maculae (d)	**S. (D.) rufispina Morawitz, 1889**
–	Femora and tibiae black (aa); wings almost uniformly dark or yellow hyaline without dark tip (bb); metasoma setae not reddish-brown (cc); one to three tergites with yellow or reddish maculae (dd)	**18**
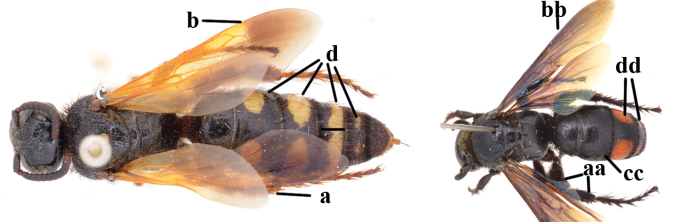		
18	T3–T4 with pair of reddish spots (a); mesonotum and pronotum posterodorsally largely impunctate (b); dorso-lateral area of propodeum impunctate on its inner half (c) [= *S. quadripustulata* auctt. from China]	**S. (D.) binotata Fabricius, 1804**
–	T3 with yellow band and T4 black (aa); mesonotum and pronotum posterodorsally densely punctate (bb) or largely impunctate; dorso-lateral area of propodeum more or less punctate (cc)	**19**
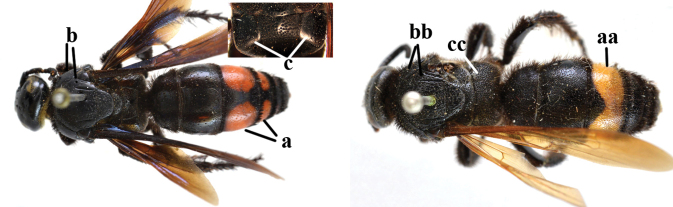		
19	Mesonotum largely impunctate; frons and vertex impunctate; frons with transverse impressed line	**20**
–	Mesonotum densely punctate (a); frons and vertex largely smooth (b) to densely punctate (bb); frons without transverse impressed line (c)	**21**
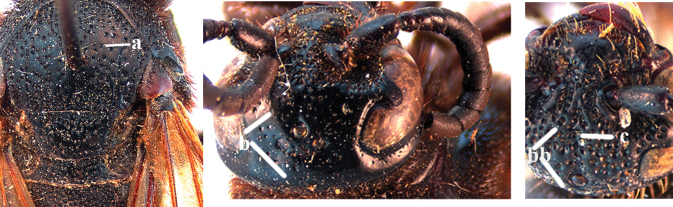		
20	Transverse impressed line on frons slightly curved upwards laterally; intervals of punctures on posterior margin of pronotum broadly smooth and polished	**S. (D.) apakaensis Tsuneki, 1972**
–	Transverse impressed line on frons straight; intervals of punctures on posterior margin of pronotum rugulose	**S. (D.) schrenckii Eversmann, 1846**
21	Head entirely black (a); median groove of frons more or less impressed (b)	**22**
–	Head partly red or yellow (aa); median groove of frons absent (bb)	**S. (D.) watanabei (Matsumura, 1912)**
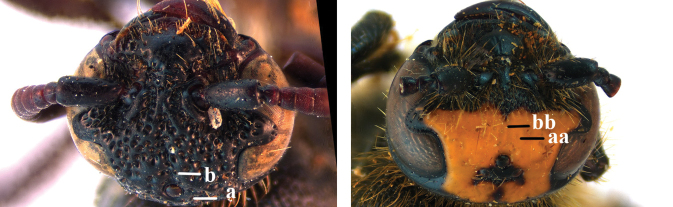		
22	Vertex and frons with dense coarse punctation (a); median groove of frons distinct (b)	**S. (D.) formosicola Betrem, 1928**
–	Vertex and frons largely smooth, without large punctures, (aa); median groove of frons indistinct (bb)	**S. (D.) oculata Matsumura, 1911**
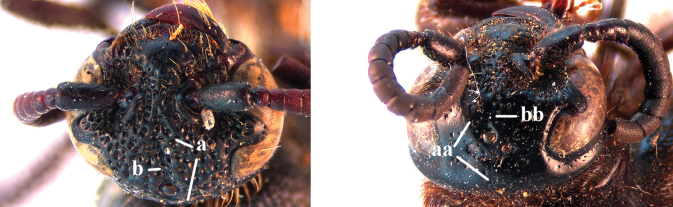		
23	Metasoma entirely black (a)	**24**
–	Metasoma with variable whitish-yellow to reddish pattern (aa)	**26**
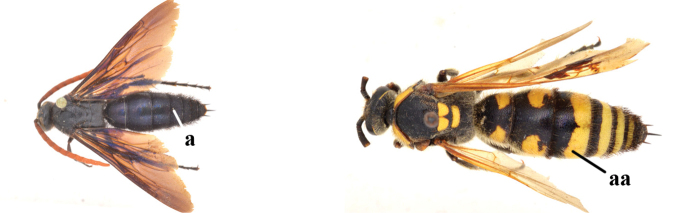		
24	Antenna robust subapically, penultimate flagellomere 1.2× longer than wide (a); anterior ocellus in a broad deep pit (b)	**S. (D.) laeviceps Smith, 1855**
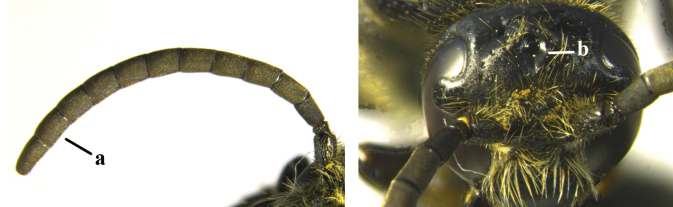		
–	Antenna slender subapically, penultimate flagellomere distinctly (1.6×) longer than wide (aa); anterior ocellus in a narrow pit (bb)	**25**
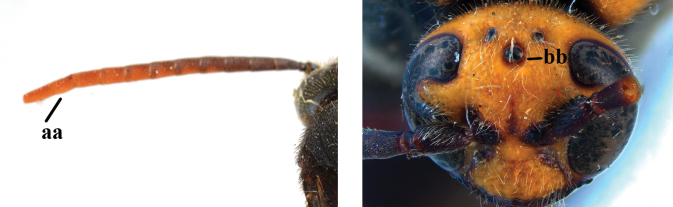		
25	Vertex entirely black (a); setae of metasoma entirely black (b)	**S. (D.) affinis Guérin-Méneville, 1845**
–	Vertex partly to nearly entirely yellowish-red (aa); setae of metasoma yellowish, but black on T6–T7 (bb)	**S. (D.) superciliaris de Saussure, 1864**
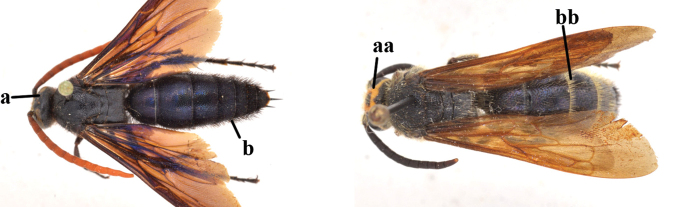		
26	Head and mesosoma entirely black (a)	**27**
–	Head and/or mesosoma with variable yellow to reddish patterns (aa)	**31**
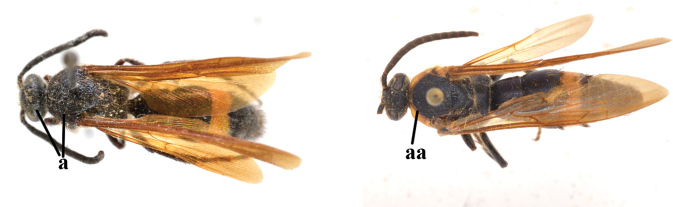		
27	Femora and tibiae red; metasoma with reddish-brown setae, even on black parts; T2–T5 with yellow band	**S. (D.) rufispina Morawitz, 1889**
–	Femora and tibiae black; setae on metasoma not reddish-brown; often only T3 with yellow or reddish band	**28**
28	Antenna shorter than head and mesosoma combined	**29**
–	Antenna longer than head and mesosoma combined (a)	**30**
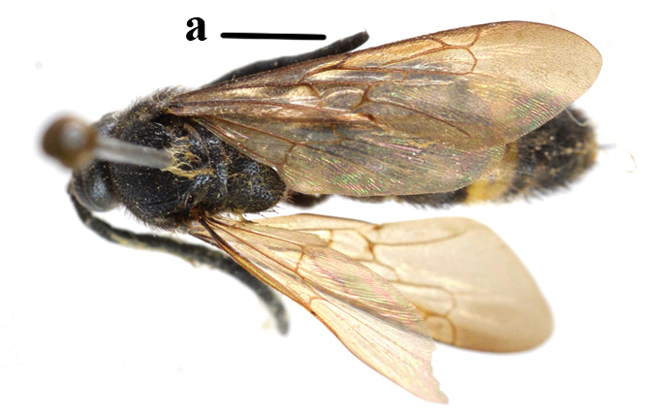		
29	Wings not paler apically; mesoscutum narrowly impunctate medio-posteriorly	**S. (D.) apakaensis Tsuneki, 1972**
–	Wings slightly paler apically; mesoscutum broadly impunctate medio-posteriorly	**S. (D.) schrenckii Eversmann, 1846**
30	Lateral areas of propodeum almost smooth (a); yellow band of T3 uninterrupted or indistinctly interrupted (b); metasoma 3.0× longer than wide (c)	**S. (D.) formosicola Betrem, 1928**
–	Lateral areas of propodeum distinctly punctate (aa); yellow band of T3 distinctly interrupted (bb); metasoma 2.4× longer than wide (cc)	**S. (D.) oculata Matsumura, 1911**
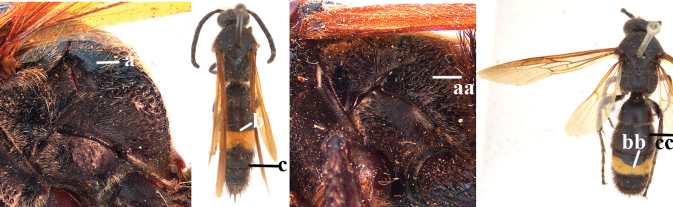		
31	Dorso-median area of propodeum smooth to superficially and sparsely punctate (intervals at least as large as puncture diameter)	**32**
–	Dorso-median area of propodeum more or less strongly and densely punctate (intervals smaller than puncture diameter)	**34**
32	Mesonotum and scutellum uniformly punctate (a); often only T3 with a more or less interrupted pale band (b)	**S. (D.) nobilis de Saussure, 1858**
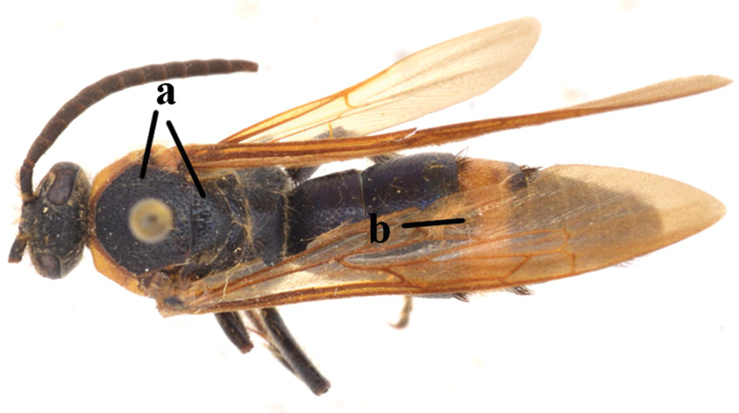		
–	Mesonotum and scutellum with impunctate area medio-posteriorly; tergites with variable pale pattern	**33**
33	T3–T5 each with a medially constricted broad band (rarely also T2 with a pair of yellow spots, yellow band on T5 sometimes greatly reduced or absent); punctures on mesosoma and propodeum (including lateral face) smaller and closer; wings brownish-yellow; punctures on metasoma smaller	**S. (D.) wusheensis Tsuneki, 1972**
–	Only T3 with two large yellow spots and T4–T5 black; punctures on mesosoma and propodeum (including lateral face) much coarser and slightly sparser; wings pale yellow; punctures on metasoma larger	**S. (D.) bnun Tsuneki, 1972**
34	Pronotum postero-dorsally yellow or red	**35**
–	Pronotum postero-dorsally black	**38**
35	Body black and reddish (a): anterior part of head largely red; frontal spatium not separated from frons (b); frons with punctures as on frontal spatium, smooth laterally (c); median groove of frons indistinct (d); T1 densely punctate, posterior margin with extremely dense and fine punctures (e)	**S. (D.) clypeata Sickman, 1894**
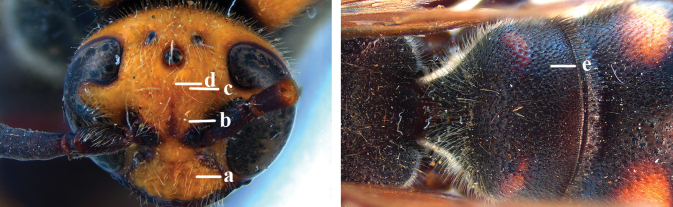		
–	Body black and yellow, head black or only with yellow spots (aa); frontal spatium usually separated from frons by a linear depression (bb), **if** not separated, then punctures on frons sparser than punctures on frontal spatium (cc); median groove of frons always distinct (dd); T1 usually sparsely punctate (ee)	**36**
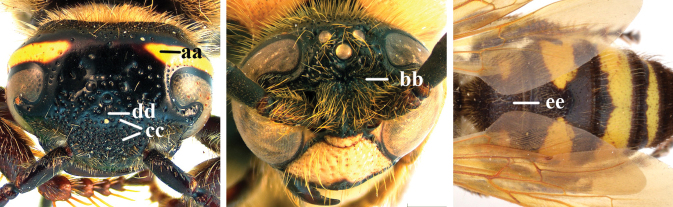		
36	Three large yellow spots present on propodeum (a); spiracular corner punctate (b)	**S. (D.) desidiosa Bingham, 1896**
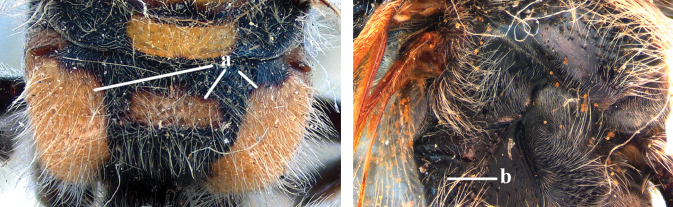		
–	Propodeum entirely black (aa); spiracular corner smooth (bb)	**37**
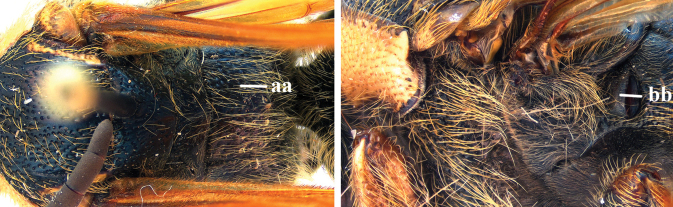		
37	Mesonotum often evenly densely punctate (a); scutellum and/or metanotum always with yellow bands (b)	**S. (D.) picteti de Saussure, 1855)**
–	Mesonotum narrowly to largely impunctate medially (aa); yellow pattern on scutellum and metanotum absent (bb) or less developed	**S. (D.) taiwana Tsuneki, 1972**
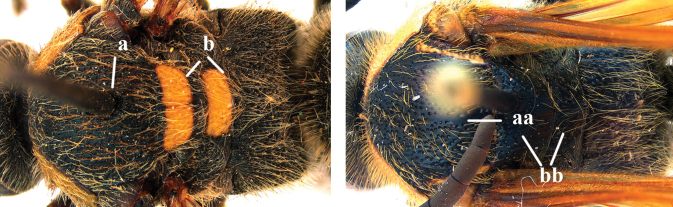		
38	T3–T4 with paired reddish spots (a); flagellum somewhat widened towards apex (b)	**S. (D.) binotata Fabricius, 1804**
–	T3 or T3–T4 with yellow bands (aa); flagellum not widened towards apex (bb)	**S. (D.) watanabei (Matsumura, 1912)**
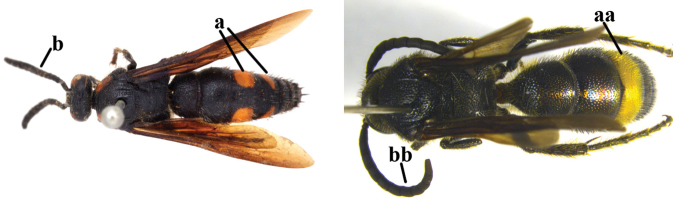		

**Notes.** The name *Scolia quadripustulata* Fabricius was misapplied for a long time to another species now known as *Scolia binotata* Fabricius (Gupta and Jonathan 2003). We checked all specimens from China in IOZ and RMNH and all were found to belong to Scolia (Discolia) binotata Fabricius. All reported records of *S. quadripustulata* from China concern *S. binotata*; therefore, we delete *S. quadripustulata* from the Chinese fauna.
